# Attitudes of Parents with Regard to Vaccination of Children against COVID-19 in Poland. A Nationwide Online Survey

**DOI:** 10.3390/vaccines9101192

**Published:** 2021-10-17

**Authors:** Mateusz Babicki, Dagmara Pokorna-Kałwak, Zbigniew Doniec, Agnieszka Mastalerz-Migas

**Affiliations:** 1 Department of Family Medicine Wroclaw Medical University, Syrokomli 1, 51-141 Wroclaw, Poland; daga_kalwak@o2.pl (D.P.-K.); agnieszka.mastalerz-migas@umed.wroc.pl (A.M.-M.); 2 Department of Pediatric Pneumonology and Allergology Institute of Tuberculosis and Lung Diseases Regional Branch, 34-700 Rabka Zdrój, Poland; zdoniec1@gmail.com; 3 Medical Institute, Podhale State College of Applied Sciences, 34-400 Nowy Targ, Poland

**Keywords:** COVID-19 vaccination, attitudes toward vaccination, COVID-19

## Abstract

Within a few months, the scientific world achieved a great success, developing effective and safe vaccines against COVID-19. Many countries with full access to vaccines have introduced recommendations for the vaccination of not only people who are at risk of developing severe COVID-19, i.e., the elderly and chronically ill, but all members of society, including children aged 12 and above as the currently registered preparations can be used above the said age. However, the use of COVID-19 vaccines in children arouses strong emotions, with their sense being frequently questioned. The aim of the paper was to assess the attitudes of Polish parents with regard to vaccinations against COVID-19 administered to their children. The study was conducted with the use of the authors’ original questionnaire, which was distributed online. The questionnaire was completed 4732 times, and 4432 surveys were qualified for the final analysis. The vast majority of the respondents were women (77.6%), people aged 36–44 (40.3%), with higher education (86.2%) and people living in the city with more than 250,000 inhabitants (48%). The mean age of the study group was 37.5 ± 6.61 years. Out of the studied group, 28.2% of parents are healthcare professionals. The study was conducted prior to the EMA’s decision that permitted the use of BNT162b2 in children. **Results:** The vast majority of the respondents were mothers, who showed significantly more favorable attitudes toward the vaccinations than fathers. Forty-four percent of parents want to vaccinate their children as soon as possible, while every fourth parent does not want to vaccinate their child at all. Main concerns about the vaccines include concerns that the preparation has not been adequately tested and that it is ineffective, as well as the lack of information concerning potential complications in the future. The main sources of information on childhood vaccinations are the media, including the Internet and television. Vaccination of the pediatric population against COVID-19 raises many emotions and doubts in parents and it is also debated by experts. The decision to vaccinate should rest on child’s parents. Both the individual benefits of protection against COVID-19 and the population benefits of pandemic control must be considered. There is a need for ongoing monitoring of the safety of administering COVID-19 vaccinations in children, as well as for evaluating their effectiveness and benefits in reducing individual risk of severe course of COVID-19 and complications after this disease, and for evaluating the population benefits of vaccines in children.

## 1. Introduction

Over a year after its declaration, the COVID-19 pandemic remains out of control. Modern medicine remains helpless against SARS-CoV-2 infection, which causes thousands of deaths in the world every day [1,2]. One of the most effective methods of protecting oneself against an infection is maintaining an adequate sanitary regime, including social distancing [3]. With time, however, there has been an increase in social backlash, which translates into reduced adherence to these rules and paves the way for the rapid spread of COVID-19 in the population [3,4,5]. This phenomenon is particularly visible among young people. Therefore, scientists focused most of their efforts on the search for effective inoculations. After months of research, information appeared about the development of the first effective and safe vaccines [6]. After the end of clinical trials, the European Medicines Agency approved preparations of various manufacturers for use [7,8,9,10]. It enabled instant implementation of population vaccination programs among adults. After a few months, Poland is at the key moment of the population vaccination program as the preparations have been approved for use in every citizen above 12 years of age (taking into account the registration records for particular preparations) [11]. In spite of that, the percentage of fully vaccinated individuals oscillates around 50%, which means that in our country it is currently below the European average [1]. Past experiences have shown that the ability to successfully vaccinate people is only half the battle. Public willingness to accept the vaccine is essential [12]. The level of vaccine acceptance among adult Poles during the pandemic remains at the level of 50%, and the percentage of people who categorically refuse to take the vaccine is at the level of 21–23% [13]. Vaccinating the highest percentage of the population possible, which will give us a chance to reach herd immunity, constitutes a great challenge for the government and health care professionals. The reduction of the transmission of the virus mainly depends on the effectiveness of the applied preparation. As statistical models show, we must inoculate 60–72% of people to achieve herd immunity, while in the case of vaccines that are 80% effective, it will be necessary to vaccinate approx. 75–90% of the population [14]. The number of people who have contracted COVID-19 should also be taken into account. However, in Poland, the data on the number of unvaccinated convalescents are not widely known. To reach the aforesaid levels, many countries with full access to the vaccines have introduced recommendations for the vaccination of not only people at risk of severe COVID-19, i.e., the elderly and chronically ill, but all members of societies, including children aged 12 and above as the currently registered preparations can be used above the said age. According to the data of the Ministry of Health from 19 July 2021, there are nearly 6.9 million people under the age of 18 in Poland, which constitutes 18.2% of the population, including 2.3 million children aged 12–17 [15]. As previous experiences have shown, administering COVID-19 vaccinations in children arouses strong emotions, and parents show extreme attitudes towards this issue [16,17,18]. Therefore, there is an urgent need to assess parents’ attitudes towards COVID-19 vaccinations, identify their major concerns, and sources of information in order to introduce effective outreach campaigns as soon as it is only possible. 

The aim of this paper was to assess the attitudes of parents towards the inoculation of children against COVID-19 as well as determine their major concerns about the vaccines. Therefore, the following research hypotheses were formulated: (1) the acceptance of childhood vaccinations against COVID-19 among parents is at a low level. (2) The age of parents as well as the number of children they have and their history of vaccinations affect their attitude towards the vaccines against COVID-19. (3) Mass media, i.e., the Internet and TV(Television) constitute the main sources of information for parents. (4) The main concerns expressed by parents include the risk of adverse events, the concern that the preparations have not been sufficiently tested in children, as well as the fear of complications in the future.

As far as we know, to date, there has been no study concerning the level of acceptance of COVID-19 vaccinations and the main concerns among parents after the implementation of population vaccinations. Similar studies were conducted before the introduction of population vaccinations, when many questions regarding the safety and efficacy of the preparations remained unanswered.

## 2. Materials and Methods

### 2.1. Methods

A CAWI (computer-assisted web interview) survey, targeted at parents of children living in Poland, was distributed online via social media. The survey was distributed mainly via the Facebook.com social network, promoting the post within groups associating parents whose topics were diverse. The survey was also disseminated in groups typically covering the subject of vaccines and SARS-CoV-2 in Poland. Another distribution channel was the Twitter.com domain. The survey was conducted from 9–14 May 2021. In this period, vaccinations were generally available to all people aged 18 and above [11]. It was not possible to vaccinate children even though, in accordance with the product characteristics, the preparation developed by Pfizer BioNTech (Pfizer, New York, NY, USA) could be administered to persons aged 16 and above [19]. Approx. 27% of the population received at least one dose of the vaccine, and 10% were fully vaccinated [1]. It was also the period when Europe was awaiting the decision of the European Medicines Agency (EMA) regarding the possibility of inoculating children aged 12–15 with the preparation developed by Pfizer BioNTech. Initial reports concerning the approval for the use of the vaccines in children aged 12–15 as well as the approval for the use of this preparation to inoculate children in Canada began to appear in the literature [20].

Before their participation in the study, the respondents were informed about the nature of the research, its methodology and objectives and, after that, informed consent was obtained from those willing to participate. There was a possibility for the participants to opt out of the study at any stage without disclosing a reason.

The study was approved by the Bioethics Committee of the Wroclaw Medical University and it was conducted in accordance with the Declaration of Helsinki (decision no. 453/2021).

The study was based on the authors’ original questionnaire that included both single and multiple-choice questions. Its first part included questions concerning informed consent and having children. If the answer to at least one of the questions was negative, the survey was terminated. The next part concerned the sociodemographic status of parents, including their age, sex, level of education and whether or not they worked in health care. Subsequently, parents were asked to enter the number of children they had as well as the age of the child whom the survey concerned. This part also included questions about the child’s chronic diseases and vaccination history, including history of adverse events. To evaluate the attitudes of parents towards the vaccinations, the authors used their original questions: “Are you planning to vaccinate your child against COVID-19?” with the following possible answers: Yes, as soon as it will be possible/Yes, but only in a few months (up to a year)/Yes, but in more than a year/I cannot decide/No, but maybe I will consider it in the future/No, never. Parents were asked to express their opinion on whether vaccinations against COVID-19 should be mandatory for children and adults. The following answers could be selected: I definitely agree/I agree/I do not agree nor disagree/I do not agree/I definitely do not agree. The probability of vaccinating the child against COVID-19 was assessed using a linear scale (1 definitely no—10 definitely yes) as was the assessment of whether the vaccines and COVID-19 were dangerous for children (1—they are safe—10 they are dangerous). Subjective perception of the level of knowledge regarding the COVID-19 vaccinations in children and the main sources of information were also assessed. 

To ensure clarity of the methodology, an English version of the questionnaire has been included in the Supplementary Materials.

### 2.2. Statistical Analysis

The statistical analysis was conducted with the use of Statistica 13.0 software, StatSoft (Statsoft, Hamburg, German). Variables were of qualitative and ordinal nature. In the case of ordinal variables, normality of distributions was assessed using three different statistical tests: Kolmogorov–Smirnov test, Lilliefors test, and Shapiro–Wilk test at the significance level of *p* = 0.05. For variables that did not meet the criterion of normality of distribution, the difference between two means was assessed using the non-parametric Mann–Whitney U test, while in the case of more variables, the Kruskal–Wallis test was performed. Correlation between quantitative variables was determined on the basis of the Spearman’s correlation analysis. The chi-square test was used to determine the relationship between the compared ordinal variables, and the value of Cramer’s V coefficient was additionally assessed.

Moreover, in the statistical analysis, a multivariate ordered logistic regression was performed. The statistically significant (*p* = 0.05) outcomes were expressed by a classical odds ratio (OR) together with a confidence 95% interval (CI 95%) and a *p*-value. In the model used, the analysis of the influence of explanatory variables such as sociodemographic variables, the parents ‘and child’s vaccination history, sources of knowledge and parents’ concerns regarding vaccination on the outcome variables: the child’s willingness to vaccinate and the probability of vaccinating the child. The multivariate ordered logistic regression was performed in R R Core Team (version 3.5.3, 2019, R Foundation for Statistical Computing, Vienna, Austria).

The significance level of *p* = 0.05 was assumed in all tests that evaluated the statistical significance of the differences between mean values. 

## 3. Results

### 3.1. Participants

Detailed distribution of the study group is presented in Table 1. 

The survey was completed 4732 times; 300 respondents did not agree to participate and/or were not parents. Final analysis included 4432 surveys. The vast majority of the respondents were women (77.6%) aged 36–44 (40.3%), with higher education (86.2%) and residing in cities of over 250,000 (48%). The mean age of the study group was 37.5 ± 6.61 years. Out of the studied group, 28.2% of parents are healthcare professionals. 

### 3.2. Personal History of Immunization and Main Sources of Information

A detailed summary of the vaccination history of the parent and child as well as primary sources of information are presented in Table 2.

Among the parents surveyed, 1389 respondents (31.4%) reported that they themselves were reluctant to be vaccinated against COVID-19, and 2409 (54.3%) had already been vaccinated. Adverse reactions to vaccines were reported by 1996 parents (70.5%), with the vast majority—1755 (87.9%)—of reactions described as mild. The vast majority of children had been so far vaccinated in accordance with the immunization schedule applicable in Poland. Two hundred and three children (4.6%) had not received any vaccinations so far. Parents of vaccinated children declared that adverse reactions to vaccines occurred in 2725 children (64.4%), with the vast predominance of mild symptoms (81.9%). The Internet is the parents’ main source of knowledge about the COVID-19 vaccines is (78.6%). Among the respondents, 78.7% declared using more than one source of knowledge.

### 3.3. Willingness to be Vaccinateed and Parents’ Main Concerns

Table 3 presents the attitudes of parents towards COVID-19 vaccinations and their main concerns about vaccinating their children. 

Among the answers of 4.432 questionnaires that were analyzed, 1955 (44.1%) parents stated were willing to vaccinate their children as soon as possible, while 1142 (25.8%) were completely opposed to the idea and did not want to vaccinate their children at all. On the linear 10-point scale assessing the likelihood of vaccinating one’s child, the mean value was 5.94 ± 3.86. The most common concerns reported by parents included the concern that the preparations have not been adequately tested (56%) and concerns about complications that may occur in the future (51.3%). In the subjective assessment of COVID-19 severity in children, the mean score was 5.09 ± 3.05.

### 3.4. Factors Affecting Willingness to Vaccinate Children

A detailed summary of the influence of sociodemographic factors, history of vaccination of parents and children, as well as the major sources of information and concerns about the vaccinations and their impact on the willingness to vaccinate one’s child are presented in Table 4 and Table 5.

Women showed significantly more favorable attitudes toward vaccinating children, as did residents of large cities. It was demonstrated that people who had encountered campaigns promoting COVID-19 vaccinations among children showed less favorable attitudes, both in the assessment of the willingness to vaccinate the child, and in the assessment on the linear scale of the likelihood of vaccinating the child.

There was a strong association between the fact that a parent was fully vaccinated and their willingness to vaccinate the child, with 79 percent of vaccinated parents wanting to vaccinate their child and as many as 71.4 percent wanting to do it as soon as possible. 

Among the major concerns associated with the inoculation, the biggest impact is visible in the case of the concern that the vaccines have not been adequately tested as well as the fear of possible complications in the future. (Cramer’s V 0.57 and 0.59,) In addition, concerns about other aspects, not addressed in this review, are strongly associated (Cramer’s V 0.60, 

There is a strong inverse correlation between the belief that the COVID-19 vaccination is unsafe for children and the willingness to vaccinate them (*r* = −0.803; *p* < 0.001). The same applies to the number of concerns about vaccination (*r* = −0.634; *p* < 0.001). A correlation between the assessment of COVID-19 severity in children and the likelihood of vaccination was also demonstrated (*r* = 0.753; *p* < 0.001).

Moreover, in the statistical analysis a multivariate ordered logistic regression was performed, the results as shown in Table 6. These results are complementary in both analyses, while in the multivariate comparison there was a reduction of statistically significant factors. There was an increase in the willingness to vaccinate with the parent’s age (OR 1.04) and the assessment of the severity of COVID-19 in children (OR 1.31). Moreover, women, health care workers, and people who have themselves been vaccinated against COVID-19 are more likely to vaccinate their children. Moreover, as the number of concerns decreases, the willingness to vaccinate their child increases (OR 0.72)

## 4. Discussion

The results of this study indicate a low level of parental acceptance regarding COVID-19 vaccination. Only 44% of parents would like to get their child vaccinated as soon as possible and 5.8% after at least a few months. At the same time, one in four parents never plans to agree to get their child vaccinated. A total of 24.3% of parents cannot decide or completely rule out getting their child vaccinated. The level of acceptance of COVID-19 vaccination for children is similar to the general attitudes of Poles towards COVID-19 vaccination [13]. With these results, many parents (40.4%) believe that vaccinations should be mandatory for children, while 51.5% of respondents are in favor of mandatory vaccination of adults. 

The results of this study are the first such reports from Poland and they are among the first in the world after the implementation of population vaccinations and the completion of studies concerning the effectiveness and safety of administering COVID-19 vaccinations in children aged 12 years and older. In earlier reports, acceptance levels of COVID-19 vaccination clarified at different levels [21,22,23]. A survey of Shenzehn factory workers in China in September 2020 showed the vaccination acceptance rate of 72.6% for their children [21]. In contrast, an Italian study conducted from December 2020 to early January 2021 showed that 60.4% of parents indicated their willingness to get their child vaccinated, while 9.9% would not do so [22]. In England there was a similar level of parental willingness to get their children vaccinated (55.8%) [23]. Those disparities can result from many aspects. Firstly, there is a different distribution period of the questionnaire, but as already mentioned, there are no reports assessing the level of parental acceptance after the implementation of population vaccinations, which shows the innovation and strength of this study. The survey distribution period is also associated with a different level of knowledge regarding COVID-19 vaccinations in both adults and children. Other important aspects include cultural conditions, the level of public confidence in authorities and the level of adaptation to government recommendations, which are particularly visible in Asian countries such as China.

Undoubtedly the parent’s gender is one of the factors that affect attitudes towards COVID-19 vaccinations. Women show more favorable attitudes towards getting their children vaccinated in both their assessment of the likelihood of COVID-19 vaccination and their desire to get their child vaccinated as soon as possible. These observations have also been proved in previous reports [22]. This is an essential aspect given that women are considered the main decision-makers regarding their children’s health [24]. It should be noted that there was an inverse relationship between the vaccination likelihood and the parent’s age with the number of children they have.

A major factor that affects the increased reluctance to get one’s own child vaccinated is the concern about the safety of the preparation used. This was proved in many previous reports, not only those related to COVID-19. A study conducted in the Philippines found a sharp reduction in administering vaccinations with a new anti-dengue preparation (Dengvaxia, (Sanofi Pasteur, Lyon, France) in response to significantly growing concerns about its safety [25]. A similar phenomenon was observed for HPV vaccination in Japan [26]. In this study, one of the strongest predictors of reluctance to COVID-19 vaccination was the parental concern about complications that may arise in the future, as well as previous experience with vaccinations, especially involving the occurrence of adverse events after the preparations used. Another important predictor of parental concern is undoubtedly the opinion of the effectiveness of the preparation used. More than 50% of parents who have doubts about the effectiveness of the used preparation will never get their children vaccinated. This phenomenon is observed both in attitudes towards COVID-19 vaccinations, where people are more likely to accept preparations with higher effectiveness, indicating that there must be a potential benefit in terms of high levels of acquired immunity compared to the level of risk taken [13,27]. There was an inverse relationship between the likelihood of getting one’s own child vaccinated and the number of concerns. Interestingly, as many as 92.8% of parents who are not afraid of anything want to get their children vaccinated as soon as possible with a probability of vaccination of 9.4 ± 1.99, proving the thesis that fear and uncertainty about the used preparation is the overriding factor limiting willingness to use it. This information should be used for implementing appropriate vaccine promotion programs with a particular emphasis on increasing parental knowledge of the safety and effectiveness of preparations used. Strangely enough, those who have been exposed to COVID-19 vaccination campaigns for children show significantly less favorable attitudes. This phenomenon may result from inadequately conducted social campaign policy—a too aggressive message, frequently arousing fear in the recipient and forcing them to a specific action and internal reflection. In some people, such an aggressive message may have the opposite effect and the recipient will be willing to ignore or repress the information obtained. Another explanation could also be the phenomenon of reactance, i.e., the pursuit of free choice. According to this psychological theory, people who feel too much compulsion to perform an activity feel like behaving in the complete opposite way [28,29]. It should also be noted that the presented study did not assess the nature of the information conveyed in social campaigns the respondents encountered, thus those campaigns might also have been negative. As is known, there have been a lot of conspiracy theories, misinformation as well as blatant antivaccine propaganda during the pandemic period. It is also a period of significant activity of the worldwide antivaccine movement, especially on the Internet [30]. The influence of the sources of vaccination information on attitudes towards getting one’s own child vaccinated should also be emphasized. Only when information was obtained from a medical doctor or from scientific reports was the highest percentage of parents willing to get their child vaccinated as soon as possible. These observations clearly indicate that the quality of provided information has a huge impact on creating human attitudes. This impact was strongly expressed in the survey, due to its methodology (CAWI).

According to the surveyed parents, COVID-19 does not pose a threat to the life and health of their child, which seems to be consistent with current medical knowledge. It is estimated that the vast majority of children with SARS-CoV-2 infection are asymptomatic or completely asymptomatic, and the death rate is as low as 0.0–0.5% depending on many factors, including the child’s age and underlying medical conditions [31,32,33,34]. A significant threat to the child’s life and health may be complications, which are described as pediatric inflammatory multisystem syndrome (PIMS), posing an immediate threat to the life of the child. Most of these complications require treatment in the intensive care unit (ICU). The estimated risk of developing PIMS is 0.1 to 1 case per 1000 children. According to scientific reports, the death rate in children in the course of PIMS oscillates around 2% [35,36,37]. Moreover, children—frequently referred to as asymptomatic carriers – may be responsible for the spread of the virus in both the household and school, representing an essential chain of viral transmission [38]. Therefore, it appears that vaccination of this age group may be a key element in breaking the chain of infection and reducing incidence rate in any age group in a significant way. This study showed the close link between COVID-19 severity scores in children and the likelihood of getting them vaccinated. These results are consistent with previous reports [23]. This is associated with the lack of proportionality between the risks taken and the benefits assessed by the parent. Opinions are also divided among experts throughout the world. As already mentioned, administering vaccinations in children aged over 12 years are allowed in Poland, similarly as in the USA, France, the Netherlands, German and Italy [39,40]. The United Kingdom are more skeptical. Initially in the UK, COVID-19 vaccinations were restricted to children (a) aged 12–15 years with comorbidities that increase the risk of the severe course of COVID-19: Down syndrome, severe neurological conditions (autism, cerebral palsy, epilepsy), immunosuppression, or (b) living with individuals at risk of the severe course of COVID-19 [41]. Nowadays, the recommendation has been verified and vaccination is recommended for each child aged 12–15 years in a single-dose schedule [42]. While COVID-19 vaccinations have been successful in protecting against severity and death among adults at risk (the elderly and chronic patients), these benefits may be less marked in children [43,44]. Firstly, children are far less likely to suffer from severe COVID-19 (e.g., the risk of hospitalization and death in the UK has been estimated at 2 per million cases) and far less likely to develop long COVID-19 syndrome [45,46]. On the other hand, vaccines reduce the spread of the virus and thus the formation of its new variants [47]. Therefore, vaccination of healthy children could have a beneficial effect on interrupting viral transmission, reducing the disease risk in a child and allowing them to safely return to school, indirectly influencing their maintenance / improvement of mental wellbeing [46]. Especially in the era of spreading the delta variant, where, according to the latest data from the USA, teenagers constitute the largest group of infected people, constituting 160.3/100,000 people, and in Melbourne, currently 45% of all infections concern children and adolescents [48,49].

There also remains the issue of worldwide disparity in access to vaccination between countries. Concern can be expressed at the ethics of using preparations intended for children whom the balance of benefits is not as significant as in groups at high risk for severe COVID-19 in countries with low access to vaccination [46]. Ethical doubts, on a global scale, are also raised by the WHO (World Health Organization): is it ethical to vaccinate children in wealthy countries with full access to vaccines, when in poorer countries this access is negligible and there is no possibility to vaccinate even risk groups?

Thus, the validity of administering COVID-19 vaccinations in children is not clear and it certainly requires further in-depth studies and observations to reach a clearer conclusion. 

The authors are aware of the limitations of this study. Undoubtedly, the method of data collection based on an anonymous, voluntary and proprietary survey distributed online via social media is a methodological limitation. Although this type of study is a safe research tool to reach a huge audience in a pandemic era, it has several limitations. These limitations include the lack of possibility to verify the truthfulness of answers, limiting the group of potential respondents to people using the Internet and social media. Moreover, due to the type of platform used, the authors are unable to determine the response rate, as well as the number of questionnaires started without completion. Another limitation of the survey is the lack of representativeness of the surveyed group for the Polish society. The overwhelming prevalence of women, individuals with a university degree and those living in large cities may significantly affect final results of the survey. However, it should be restated that women—mothers—are usually main decision-makers regarding their child’s health, which significantly affected the disproportionate number of female respondents [24]. In addition, in the future, the questionnaire should be extended to include questions about the presence of specific chronic diseases, the past COVID-19 of parents and loved ones, as well as the type of preparation that the parent was vaccinated with, because these variables may also affect the final decision regarding vaccination of the child [13].

In conclusion, the results of this study indicate a moderate level of public acceptance of COVID-19 vaccination for children in Poland. The main predictors of reluctance to COVID-19 vaccination are concerns about lack of adequate testing, effectiveness and uncertainty about their impact on children’s future life. 

After one month of accepting vaccination of children aged 12 years and older in Poland, at least one dose was given to 25% of them, and the immunization schedule was completed in almost 16% [15]. This result is far below the expectations and declarations of parents regarding getting their child of this age group vaccinated as soon as possible. This unfortunately indicates a disturbing trend of limited trust and thus less willingness to get one’s own child vaccinated.

## 5. Conclusions

Vaccination of the pediatric population against COVID-19 raises many emotions and doubts in parents and it is also debated by experts. The decision to vaccinate should rest on the child’s parents. Both the individual benefits of protection against COVID-19 and the population benefits of pandemic control must be considered.

There is a need for ongoing monitoring of the safety of administering COVID-19 vaccinations in children, as well as for evaluating their effectiveness and benefits in reducing individual risk of severe course of COVID-19 and complications after this disease, and for evaluating the population benefits of vaccines in children. 

As the most common source of information, mass media can be a safe and effective way to reach as many people as possible with reliable knowledge in a short period of time. The biggest challenge of vaccination awareness campaigns, however, is to develop effective methods to combat infodemia and fake news.

## Figures and Tables

**Table 1:** Characteristics of the study group.

Variable		N(%)
Sex	Female	3440 (77.6)
	Male	993 (22.4)
Age	18–29	436 (9.9)
	30–35	150 (32.7)
	36–44	1788 (40.3)
	>45	759 (17.1)
Place of residence	Rural area	777 (17.5)
	City of up to 50,000 inhabitants	681 (15.4)
	City of 50,000–250,000 inhabitants	848 (19.1)
	City with more than 250,000 inhabitants	2126 (48.0)
Education	Higher	3823 (86.2)
	Secondary education	532 (12.0)
	Vocational	48 (1.1)
	Others	30 (0.7)
Healthcare professional	Yes	1251 (28.2)
	No	3182 (71.8)
Vaccination against COVID-19	Yes	2409 (54.3)
	No, but I am waiting to be vaccinated	635 (14.3)
	No, I do not want to get vaccinated	1389 (31.4)
Number of children	1	1685 (38)
	2	1973 (44.5)
	3	587 (13.2)
	4	107 (2.4)
	5	43 (1.0)
	>5	38 (0.9)
How old is your child?	<6 months	155 (3.5)
	6–23 months	579 (13.1)
	2–11 years	2509 (56.6)
	12–18 years	1189 (26.8)
Child suffers from chronic diseases	Yes	573 (12.9)
	No	3860 (87.1)
Have you seen the campaign concerning vaccinations against COVID-19 in adults?	Yes	3897 (87.9)
	No	536 (12.1)
Have you seen the campaign concerning vaccinations against COVID-19 in children?	Yes	1153 (26.1)
	No	3280 (73.9)

**Table 2:** Parent’s and child’s history of immunization and assessment of main sources of information on COVID-19 vaccines.

Question	Variable	N (%)
COVID-19 vaccination received by parent (n = 4433)	Yes	2409 (54.3)
	Yes, I am waiting to be vaccinated	635 (14.3)
	No, I do not want to get vaccinated	1389 (31.4)
Adverse reaction after vaccination (n = 2831)	Yes, severe	23 (0.9)
	Yes, moderate	218 (7.7)
	Yes, mild	1755 (61.9)
	No	835 (29.5)
Previous vaccinations in child (n = 4433)	Yes, mandatory and recommended	2905 (65.5)
	Yes, mandatory	1325 (29.9)
	No	203 (4.6)
Adverse reactions after vaccination in child (n = 4230)	Yes, severe	91 (2.2)
	Yes, moderate	400 (9.4)
	Yes, mild	2234 (52.8)
	No	1505 (35.6)
Sources of knowledge on COVID-19 vaccinations used by parents		
Internet		3486 (78.6)
Television		776 (17.5)
Physician		1917 (43.2)
Medical professional other than physician		1243 (28.0)
Scientific reports		2554 (57.6)
Patient information leaflets		736 (16.6)
Friends (who are not medical professionals)		564 (12.7)
Other		709 (15.9)
Average number of sources of knowledge *		2.7 ± 1.37
Subjective sense of level of knowledge about vaccinations **		6.56 ± 2.74

* mean ± standard deviation; ** mean ± standard deviation on a 10-point scale.

**Table 3:** Parents’ attitudes toward COVID-19 vaccinations in children with regard to mandatory vaccinations, and major concerns about vaccinations in children.

Question	Variable	N (%)
Are you planning to vaccinate your child?	Yes, as soon as possible	1955 (44.1)
	Yes, but in a few months (up to a year)	212 (4.8)
	Yes, but in a year or more	46 (1.0)
	I cannot decide	502 (11.3)
	No, but I might consider it in the future	576 (13.0)
	No, never	142 (25.8)
Probability of vaccinating the child *		5.94 ± 3.86
Do you think vaccinations should be mandatory for children?	Definitely yes	968 (21.9)
	Rather yes	822 (18.5)
	Neither yes nor no	593 (13.4)
	Rather no	391 (8.8)
	Definitely no	1659 (37.4)
Do you think vaccinations should be mandatory in adults?	Definitely yes	1514 (34.2)
	Rather yes	768 (17.3)
	Neither yes nor no	309 (7.0)
	Rather no	300 (6.7)
	Definitely no	1542 (34.8)
Parents’ concerns related to vaccinating their children against COVID-19		
Adverse reaction to vaccination	Yes	2095 (47.3)
Lack of adequate testing of the preparation	Yes	2482 (56.0)
Incorrect transport/storage	Yes	712 (16.1)
Lack of effectiveness	Yes	1043 (23.5)
Complications in the future	Yes	2278 (51.3)
Other	Yes	343 (7.7)
No concerns	Yes	846 (19.1)
Assessment of the risk of COVID-19 vaccination for the child *		5.15 ± 3.31
Assessment of COVID-19 severity among children *		5.09 ± 3.05

* mean ± standard deviation on a 10-point scale.

**Table 4:** The impact of sociodemographic factors on attitudes towards vaccinations in children.

Variable		Willingness to Vaccinate N(%)						*p*	Cramér’s V	Probability of Vaccinating Child M (SD)	*p*
		As Soon as Possible	Yes, in a Few Months	Yes but in a Year or More	I Cannot Decide	No but I Might Consider it in the Future	No, Never				
Sex	Female	1707 (49.6)	200 (5.8)	41 (1.2)	456 (13.3)	395 (11.5)	641 (18.6)	<0.001	0.34	6.59 ± 3.65	<0.001
	Male	248 (25.0)	12 (1.2)	5 (0.5)	46 (4.6)	181 (18.2)	501 (50.5)			3.72 ± 3.72	
Age	18–29	167 (38.3)	28 (6.4)	8 (1.8)	72 (16.5)	72 (16.5)	89 (20.5)	<0.001	0.35	6.03 ± 3.57	<0.001
	30–35	686 (47.3)	84 (5.8)	18 (1.2)	224 (15.5)	181 (12.5)	257 (17.7)			6.43 ± 3.58	
	36–44	773 (43.2)	76 (4.3)	17 (1.0)	173 (9.8)	222 (12.3)	527 (29.4)			5.77 ± 3.97	
	>45	329 (43.4)	24 (3.2)	3 (0.4)	33 (4.4)	101 (13.2)	269 (35.4)			5.39 ± 4.17	
Place of residence	Rural area	309 (39.8)	33 (4.2)	9 (1.2)	104 (13.4)	99 (12.7)	223 (28.7)	<0.001	0.07	5.64 ± 3.83	<0.001
	City < 50,000 inhabitants	263 (38.6)	34 (5.0)	6 (0.9)	86 (12.6)	85 (12.5)	207 (30.4)			5.51 ± 3.87	
	City 50,000–250,000 inhabitants	305 (36.0)	41 (4.8)	7 (0.8)	114 (13.4)	121 (14.3)	260 (30.7)			5.35 ± 3.85	
	City > 250,000 inhabitants	1078 (50.7)	104 (4.9)	24 (1.1)	198 (9.3)	271 (12.8)	451 (21.2)			6.43 ± 3.81	
Healthcare professional	Yes	859 (68.6)	67 (5.4)	12 (1.0)	84 (6.7)	105 (8.4)	124 (9.9)	<0.001	0.33	7.91 ± 3.19	<0.001
	No	1096 (34.4)	145 (4.6)	32 (1.1)	418 (13.1)	471 (14.8)	1018 (32.0)			5.18 ± 3.82	
Number of children	1	795 (47.2)	96 (5.7)	25 (1.5)	221 (13.1)	208 (12.3)	340 (20.2)	<0.001	0.08	6.35 ± 3.70	<0.001
	2	867 (43.9)	85 (4.3)	17 (0.9)	219 (11.1)	270 (13.7)	515 (26.1)			5.90 ± 3.87	
	3	239 (40.7)	28 (4.8)	3 (0.5)	54 (9.2)	67 (11.4)	196 (33.4)			5.50 ± 4.02	
	4	33 (30.8)	0 (0.0)	0 (0.0)	7 (6.6)	20 (18.7)	47 (43.9)			4.42 ± 4.01	
	5	13 (30.2)	2 (4.7)	0 (0.0)	1 (2.3)	4 (9.3)	23 (53.5)			4.18 ± 4.14	
	>5	8 (21.1)	1 (2.6)	1 (2.6)	0 (0.0)	7 (18.4)	21 (55.3)			3.45 ± 3.70	
How old is your child?	<6 months	67 (43.2)	7 (4.5)	4 (2.6)	20 (12.9)	25 (16.1)	32 (20.7)	<0.001	0.08	6.06 ± 3.73	<0.001
	6–23 months	242 (41.8)	39 (6.7)	8 (1.4)	112 (19.3)	82 (14.2)	96 (16.6)			6.34 ± 3.48	
	2–11 years	1095 (43.6)	124 (4.9)	25 (1.0)	291 (11.7)	319 (12.7)	655 (26.1)			5.90 ± 3.83	
	12–18 years	550 (46.3)	42 (3.5)	9 (0.8)	79 (6.6)	150 (12.6)	359 (30.2)			5.82 ± 4.09	
Child suffers from chronic diseases	Yes	282 (49.2)	26 (4.5)	7 (1.2)	68 (11.9)	68 (11.9)	122 (21.3)	0.072	0.05	6.43 ± 3.74	0.002
	No	1673 (43.4)	186 (4.8)	39 (1.0)	434 (11.2)	508 (13.2)	1020 (26.4)			5.88 ± 3.87	
COVID-19 vaccination campaign for adults	Yes	1731 (44.4)	187 (4.8)	40 (1.0)	429 (11.0)	493 (12.7)	1017 (26.1)	0.158	0.04	5.96 ± 3.87	0.522
	No	224 (41.8)	25 (4.7)	6 (1.1)	73 (13.6)	83 (15.5)	125 (23.3)			5.82 ± 3.78	
COVID-19 vaccination campaign for children	Yes	371 (32.2)	34 (3.0)	6 (0.5)	56 (4.8)	136 (11.8)	550 (47.7)	<0.001	0.31	4.43 ± 4.03	<0.001
	No	1584 (48.3)	178 (5.4)	40 (1.2)	446 (13.6)	440 (13.4)	592 (18.1)			6.48 ± 4.44	

**Table 5:** The impact of one’s own vaccination history, sources of information and major concerns on attitudes towards getting one’s own children vaccinated against COVID-19.

Variable		Willingness to Vaccination N(%)						*p*	Cramér’s V	Probability of Vaccination M (SD)	*p*
		As Soon as Possible	Yes but Only in a Few Months	Yes but in a Year or More	I Cannot Decide	No but I Might Consider it in the Future	No, Never				
COVID-19 vaccination received by parent	Yes	1721 (71.4)	157 (6.5)	26 (1.1)	304 (12.7)	150 (6.2)	51 (2.1)	<0.001	0.64	8.44 ± 2.44	<0.001
	No, I’m waiting for a vaccination	232 (36.6)	53 (8.4)	15 (2.5)	177 (27.9)	121 (19.1)	37 (5.8)			6.43 ± 3.03	
	No, I don’t want to get vaccinated	2 (0.1)	2 (0.1)	5 (0.4)	21 (1.5)	305 (22.0)	1054 (75.9)			1.40 ± 1.18	
Adverse events after COVID-19 vaccination in parents	Yes, severe	3 (13.0)	0 (0.0)	0 (0.0)	0 (0.0)	1 (4.4)	19 (82.6)	<0.001	0.31	2.17 ± 3.10	<0.001
	Yes, moderate	109 (50.0)	14 (6.4)	2 (0.9)	35 (16.1)	27 (12.4)	31 (14.2)			6.81 ± 3.52	
	Yes, mild	1263 (72.0)	118 (6.7)	23 (1.3)	223 (12.7)	95 (5.4)	33 (1.9)			8.50 ± 2.37	
	No	381 (45.6)	32 (3.8)	4 (0.5)	79 (9.5)	106 (12.7)	233 (27.9)			5.93 ± 3.95	
History of vaccinations in child	Yes, mandatory and recommended	1730 (59.5)	174 (6.0)	40 (1.4)	363 (12.5)	306 (10.5)	292 (10.1)	<0.001	0.40	7.42 ± 3.30	<0.001
	Yes, mandatory	213 (16.1)	37 (2.8)	6 (0.4)	233 (10.0)	253 (19.1)	683 (51.6)			3.31 ± 3.31	
	No	12 (5.9)	1 (0.5)	0 (0.0)	6 (3.0)	17 (8.4)	167 (82.2)			2.03 ± 2.51	
Adverse reactions in child after previous vaccinations	Yes, severe	17 (12.3)	3 (2.3)	1 (0.7)	5 (3.6)	13 (9.4)	99 (71.7)	<0.001	0.15	2.56 ± 3.15	<0.001
	Yes, moderate	122 (27.2)	28 (6.2)	6 (1.3)	44 (9.8)	299 (14.5)	184 (41.0)			4.58 ± 3.83	
	Yes, mild	1117 (49.4)	126 (5.6)	28 (1.2)	284 (12.6)	299 (13.2)	407 (18.0)			6.51 ± 3.67	
	No	699 (44.1)	55 (3.5)	11 (0.7)	169 (10.7)	199 (12.6)	452 (28.4)			5.82 ± 3.93	
Sources of knowledge											
Internet	Yes	1470 (42.2)	175 (5.0)	40 (1.2)	445 (12.8)	476 (13.6)	880 (25.2)	<0.001	0.11	5.88 ± 3.80	<0.001
	No	485 (51.2)	37 (3.9)	6 (0.6)	57 (6.0)	100 (10.6)	262 (27.7)			6.21 ± 4.03	
Television	Yes	271 (34.9)	31 (4.0)	6 (0.8)	130 (16.7)	145 (18.7)	193 (24.9)	<0.001	0.12	5.33 ± 3.68	<0.001
	No	1684 (46.1)	181 (5.0)	40 (1.1)	372 (10.2)	431 (11.8)	949 (25.8)			6.08 ± 3.88	
Physician	Yes	779 (40.6)	96 (5.0)	21 (1.1)	214 (11.2)	275 (14.4)	532 (27.7)	0.001	0.07	5.67 ± 3.88	<0.001
	No	1176 (36.7)	116 (4.6)	25 (1.0)	288 (11.5)	301 (11.9)	610 (24.3)			6.15 ± 3.82	
Medical professional other than physician	Yes	472 (38.0)	60 (4.8)	10 (0.8)	145 (11.7)	189 (15.2)	367 (29.5)	<0.001	0.08	5.47 ± 3.86	<0.001
	No	1483 (46.5)	152 (4.8)	36 (1.1)	357 (11.2)	387 (12.1)	775 (24.3)			6.13 ± 3.84	
Scientific reports	Yes	1192 (46.7)	113 (4.4)	27 (1.1)	163 (6.4)	194 (11.5)	765 (29.9)	<0.001	0.21	5.94 ± 4.02	0.746
	No	763 (40.6)	99 (5.3)	19 (1.0)	339 (18.0)	282 (15.0)	377 (20.1)			5.95 ± 3.62	
Patient information leaflets	Yes	284 (38.6)	35 (4.8)	9 (1.2)	80 (10.9)	106 (14.4)	222 (30.1)	0.013	0.06	5.48 ± 3.90	<0.001
	No	1671 (45.2)	177 (4.8)	37 (1.0)	422 (11.4)	470 (12.7)	920 (24.9)			6.03 ± 3.84	
Friends (who are not medical professionals)	Yes	135 (23.9)	29 (5.1)	3 (0.5)	90 (16.0)	99 (17.6)	208 (36.9)	<0.001	0.16	4.47 ± 3.62	<0.001
	No	1820 (47.0)	183 (4.7)	43 (1.1)	412 (10.7)	477 (12.3)	934 (24.1)			6.16 ± 3.84	
Other	Yes	210 (29.6)	18 (2.5)	4 (0.7)	53 (7.5)	81 (11.3)	343 (48.4)	<0.001	0.23	4.43 ± 3.91	<0.001
	No	1745 (46.9)	194 (5.2)	42 (1.1)	449 (12.1)	495 (13.3)	799 (21.5)			6.26 ± 3.76	
Mandatory vaccinations against COVID-19											
Do you think vaccinations should be mandatory for children?	Definitely yes	937 (96.8)	25 (2.6)	1 (0.1)	4 (0.4)	0 (0.0)	1 (0.1)	<0.001	0.53	9.82 ± 0.63	<0.001
	Rather yes	650 (79.1)	93 (11.3)	12 (1.5)	64 (7.8)	3 (0.4)	0 (0.0)			9.04 ± 1.28	
	Neither yes nor no	234 (39.5)	60 (10.1)	12 (2.0)	242 (40.8)	44 (7.4)	1 (0.2)			7.17 ± 2.32	
	Rather no	105 (26.9)	29 (7.4)	11 (2.8)	128 (32.7)	114 (29.2)	4 (1.0)			6.07 ± 2.71	
	Definitely no	29 (1.8)	5 (0.3)	10 (0.6)	64 (3.9)	415 (25.0)	1136 (68.4)			1.68 ± 1.67	
Do you think vaccinations should be mandatory in adults?	Definitely yes	1238 (81.8)	91 (6.0)	11 (0.7)	138 (9.1)	32 (2.1)	4 (0.3)	<0.001	0.47	9.16 ± 1.66	<0.001
	Rather yes	470 (61.2)	71 (9.2)	15 (1.9)	164 (21.5)	47 (6.1)	1 (0.1)			8.12 ± 2.21	
	Neither yes nor no	137 (44.3)	29 (9.5)	5 (1.6)	86 (27.8)	43 (13.9)	9 (2.9)			6.88 ± 2.83	
	Rather no	83 (27.7)	19 (6.3)	7 (2.3)	74 (24.7)	102 (34.0)	15 (5.0)			5.60 ± 3.04	
	Definitely no	27 (1.8)	2 (0.1)	8 (0.5)	40 (2.6)	352 (22.8)	1113 (72.2)			1.59 ± 1.62	
Concerns about vaccinations											
Adverse reaction to vaccination	Yes	540 (25.8)	96 (4.7)	16 (0.8)	287 (13.6)	349 (16.7)	807 (38.4)	<0.001	0.37	4.41 ± 3.64	<0.001
	No	1415 (60.5)	116 (5.0)	30 (1.3)	215 (9.2)	227 (9.7)	335 (14.3)			7.32 ± 3.50	
Lack of adequate testing of the preparation	Yes	474 (19.1)	146 (5.9)	39 (1.6)	418 (16.8)	498 (20.1)	907 (36.5)	<0.001	0.57	4.17 ± 3.39	<0.001
	No	1481 (75.9)	66 (3.3)	7 (0.4)	84 (4.3)	78 (4.0)	235 (12.1)			8.21 ± 3.17	
Incorrect transport/storage	Yes	259 (36.4)	30 (4.2)	9 (1.3)	79 (11.1)	99 (13.8)	236 (33.2)	<0.001	0.08	5.23 ± 3.89	<0.001
	No	1696 (45.6)	182 (4.9)	37 (1.0)	423 (11.4)	477 (12.7)	906 (24.4)			6.08 ± 3.84	
Lack of effectiveness	Yes	211 (20.2)	25 (2.4)	6 (0.6)	50 (4.8)	181 (17.4)	570 (54.6)	<0.001	0.40	3.41 ± 3.55	<0.001
	No	1744 (51.5)	187 (5.5)	40 (1.2)	452 (13.3)	395 (11.6)	572 (16.9)			6.73 ± 3.61	
Complications in the future	Yes	380 (16.7)	99 (4.3)	24 (1.0)	341 (15.0)	471 (20.7)	963 (42.3)	<0.001	0.59	3.77 ± 3.33	<0.001
	No	1575 (73.1)	113 (5.2)	22 (1.0)	161 (7.5)	105 (4.9)	179 (8.3)			8.26 ± 2.91	
Other	Yes	50 (14.6)	5 (1.4)	1 (0.3)	11 (3.2)	63 (18.4)	213 (62.1)	<0.001	0.26	2.76 ± 3.14	<0.001
	No	1905 (46.6)	207 (5.1)	45 (1.1)	491 (12.0)	513 (12.5)	929 (22.7)			6.22 ± 3.79	
No concerns	Yes	785 (92.8)	8 (1.0)	2 (0.2)	5 (0.6)	9 (1.1)	37 (4.4)	<0.001	0.48	9.40 ± 1.99	<0.001
	No	1170 (32.6)	204 (5.7)	44 (1.2)	497 (13.9)	567 (15.8)	1105 (30.8)			5.13 ± 3.74	

**Table 6:** Multivariate ordered logistic regression taking into account sociodemographic, one’s own vaccination history, sources of information and major concerns on attitudes towards getting one’s own children vaccinated against COVID-19.

	Are You Planning to Vaccinate Your Child?			Probability of Vaccinating the Child		
Risk factors	OR	CI (95%)	*p*-value	OR	CI (95%)	*p*-value
Sex, female	---	----	---	0.68	(0.53–0.87)	0.0010
Parent’s age, year	1.04	(1.02–1.07)	<0.0001	1.04	(1.03–1.06)	<0.0001
Healthcare professional	1.46	(1.17–1.84)	0.0091	1.47	(1.22–1.81)	0.0003
Adverse reaction after parents COVID-19 vaccination	0.73	(0.60–0.88)	0.0012	0.82	(0.69–0.96)	0.0150
History of vaccinations in child	1.93	(1.56–2.40)	<0.0001	1.67	(1.38–2.02)	<0.0001
Mandatory vaccinations for a child	3.68	(3.19–4.25)	<0.0001	3.10	(2.76–3.48)	<0.0001
Mandatory vaccinations for adults	0.80	(0.71–0.91)	0.0001	0.85	(0.75–0.91)	0.0034
COVID-19 vaccination campaign for children	----	----	----	0.81	(0.72–0.90)	0.0002
Assessment of COVID-19 severity among children	1.31	(1.25–1.38)	<0.0001	1.26	(1.21–1.38)	<0.0001
Assessment of the risk of COVID-19 vaccination for the child	0.68	(0.65–0.71)	<0.0001	0.73	(0.70–0.76)	<0.0001
Parents’ concerns, lack of adequate testing of the preparation	0.45	(0.35–0.57)	<0.0001	0.66	(0.52–0.83)	0.0003
No concerns	5.12	(3.14–8.34)	<0.0001	3.59	(2.55–5.03)	<0.0001
Number of concerns	0.84	(0.73–0.95)	0.0071	0.72	(0.63–0.82)	<0.0001
COVID-19 vaccination received by the parent						
No, I’m waiting for a vaccination vs No, I don’t want to get vaccinated	2.73	(1.54–4.85)	0.0006	3.90	(2.28–6.68)	<0.0001
No, I don’t want to get vaccinated vs Yes, I’m vaccinated	8.51	(5.21–13.91)	<0.0001	9.03	(5.70–14.29)	<0.0001

## Data Availability

Not applicable.

## Supplementary Materials

The following are available online at https://www.mdpi.com/article/10.3390/vaccines9101192/s1 Survey.

## References

[1] Ritchie H., Ortiz-Ospina E., Beltekian D., Mathieu E., Hasell J., Macdonald B., Giattino C., Appel C., Rodés-Guirao L., Roser M. (2020). Coronavirus Pandemic (COVID-19). Our World Data.

[2] Flisiak R., Horban A., Jaroszewicz J., Kozielewicz D., Mastalerz-Migas A., Owczuk R., Parczewski M., Pawłowska M., Piekarska A., Simon K. (2021). Management of SARS-CoV-2 infection: Recommendations of the Polish Association of Epidemiologists and Infectiologists as of 26 April 2021. Pol. Arch. Intern. Med..

[3] de Souza Melo A., da Penha Sobral A.I.G., Marinho M.L.M., Duarte G.B., Vieira A.A., Sobral M.F.F. (2021). The impact of social distancing on COVID-19 infections and deaths. Trop. Dis. Travel Med. Vaccines.

[4] Bloomberg. https://www.bloomberg.com/opinion/articles/2020-11-14/2020-s-covid-protests-are-a-sign-of-the-social-unrest-to-come.

[5] Money L., Fry H., Lai S., Lin R.-G. (2020). A Revolt Against Wearing Masks Creates a New Coronavirus Danger as California Reopens. Los Angeles Times.

[6] Pfizer oraz BioNTech Pracują nad Rozwojem Potencjalnej Szczepionki na COVID-19. https://www.pfizer.com.pl/o-firmie/press-room/pfizer-oraz-biontech-pracuj%C4%85-nad-rozwojem-potencjalnej-szczepionki-na-covid-19.

[7] https://www.ema.europa.eu/en/news/ema-recommends-COVID-19-vaccine-astrazeneca-authorisation-eu.

[8] https://www.ema.europa.eu/en/news/ema-recommends-COVID-19-vaccine-moderna-authorisation-eu.

[9] https://www.ema.europa.eu/en/news/ema-recommends-first-COVID-19-vaccine-authorisation-eu.

[10] https://www.ema.europa.eu/en/news/ema-recommends-COVID-19-vaccine-janssen-authorisation-eu.

[11] Zarejestruj Się na Szczepienie Przeciw COVID-19—Szczepienie Przeciwko COVID-19—Portal Gov.pl. https://www.gov.pl/web/szczepimysie/rejestracja.

[12] Neumann-Böhme S., Varghese N.E., Sabat I., Barros P.P., Brouwer W., van Exel J., Schreyögg J., Stargardt T. (2020). Once we have it, will we use it? A European survey on willingness to be vaccinated against COVID-19. Eur. J. Health Econ..

[13] Babicki M., Mastalerz-Migas A. (2021). Attitudes toward vaccination against COVID-19 in Poland. A longitudinal study performed before and two months after the commencement of the population vaccination programme in Poland. Vaccines (Basel).

[14] Anderson R.M., Vegvari C., Truscott J., Collyer B.S. (2020). Challenges in creating herd immunity to SARS-CoV-2 infection by mass vaccination. Lancet.

[15] GUS Ludność. Stan i Struktura Ludności oraz ruch Naturalny w Przekroju Terytorialnym (Stan W Dniu 31.12.2020). https://stat.gov.pl/obszary-tematyczne/ludnosc/ludnosc/ludnosc-stan-i-struktura-ludnosci-oraz-ruch-naturalny-w-przekroju-terytorialnym-stan-w-dniu-31-12-2020,6,29.html.

[16] Stefanoff P., Mamelund S.-E., Robinson M., Netterlid E., Tuells J., Bergsaker M.A.R., Heijbel H., Yarwood J. (2010). VACSATC working group on standardization of attitudinal studies in Europe Tracking parental attitudes on vaccination across European countries: The Vaccine Safety, Attitudes, Training and Communication Project (VACSATC). Vaccine.

[17] Hadjipanayis A., van Esso D., Del Torso S., Dornbusch H.J., Michailidou K., Minicuci N., Pancheva R., Mujkic A., Geitmann K., Syridou G. (2020). Vaccine confidence among parents: Large scale study in eighteen European countries. Vaccine.

[18] Pisaniak P., Konarska M., Tarczon A., Stawowy B., Bejster K., Piórek W., Mędrzycka-Dąbrowska W., Ozga D. (2021). Mothers’ opinions on vaccinations and penal responsibility for vaccination avoidance in nine selected European countries: Findings from a cross-sectional survey. Risk Manag. Healthc. Policy.

[19] Ulotka & ChPL Comirnaty. https://www.pfizerpro.com.pl/product/comirnaty/szczepionka-mrna-przeciw-covid-19/ulotka-chpl-comirnaty.

[20] Office of the Commissioner Coronavirus (COVID-19) Update: FDA Authorizes Pfizer-BioNTech COVID-19 Vaccine for Emergency Use in Adolescents in Another Important Action in Fight against Pandemic. https://www.fda.gov/news-events/press-announcements/coronavirus-covid-19-update-fda-authorizes-pfizer-biontech-covid-19-vaccine-emergency-use.

[21] Zhang K.C., Fang Y., Cao H., Chen H., Hu T., Chen Y.Q., Zhou X., Wang Z. (2020). Parental acceptability of COVID-19 vaccination for children under the age of 18 years: Cross-sectional online survey. JMIR Pediatr. Parent..

[22] Montalti M., Rallo F., Guaraldi F., Bartoli L., Po G., Stillo M., Perrone P., Squillace L., Dallolio L., Pandolfi P. (2021). Would parents get their children vaccinated against SARS-CoV-2? Rate and predictors of vaccine hesitancy according to a survey over 5000 families from Bologna, Italy. Vaccines (Basel).

[23] Bell S., Clarke R., Mounier-Jack S., Walker J.L., Paterson P. (2020). Parents’ and guardians’ views on the acceptability of a future COVID-19 vaccine: A multi-methods study in England. Vaccine.

[24] Role of Mothers in Assuring Children Receive COVID-19 Vaccinations. https://www.kff.org/womens-health-policy/issue-brief/role-of-mothers-in-assuring-children-receive-covid-19-vaccinations/.

[25] Larson H.J. (2018). Politics and public trust shape vaccine risk perceptions. Nat. Hum. Behav..

[26] Larson H.J., Wilson R., Hanley S., Parys A., Paterson P. (2014). Tracking the global spread of vaccine sentiments: The global response to Japan’s suspension of its HPV vaccine recommendation. Hum. Vaccines Immunother..

[27] Harapan H., Wagner A.L., Yufika A., Winardi W., Anwar S., Gan A.K., Setiawan A.M., Rajamoorthy Y., Sofyan H., Mudatsir M. (2020). Acceptance of a COVID-19 vaccine in southeast Asia: A cross-sectional study in Indonesia. Front. Public Health.

[28] Buglewicz K. (2015). Kampanie społeczne—Idea vs biznes. Zesz. Prasozn..

[29] Tokarczyk E. (2013). Kampanie społeczne w systemie działań na rzecz bezpieczeństwa ruchu drogowego. AUTOBUSY Tech. Eksploat. Syst. Transp..

[30] Johnson N.F., Velásquez N., Restrepo N.J., Leahy R., Gabriel N., El Oud S., Zheng M., Manrique P., Wuchty S., Lupu Y. (2020). The online competition between pro- and anti-vaccination views. Nature.

[31] Bhopal S.S., Bagaria J., Olabi B., Bhopal R. (2021). Children and young people remain at low risk of COVID-19 mortality. Lancet Child Adolesc. Health.

[32] Patel N.A. (2020). Pediatric COVID-19: Systematic review of the literature. Am. J. Otolaryngol..

[33] Zimmermann P., Curtis N. (2020). Coronavirus infections in children including COVID-19: An overview of the epidemiology, clinical features, diagnosis, treatment and prevention options in children. Pediatr. Infect. Dis. J..

[34] Children and COVID-19: State-Level Data Report. https://www.aap.org/en/pages/2019-novel-coronavirus-covid-19-infections/children-and-covid-19-state-level-data-report/.

[35] Okarska-Napierała M., Ludwikowska K.M., Szenborn L., Dudek N., Mania A., Buda P., Książyk J., Mazur-Malewska K., Figlerowicz M., Szczukocki M. (2020). Pediatric inflammatory multisystem syndrome (PIMS) did occur in Poland during months with low COVID-19 prevalence, preliminary results of a nationwide register. J. Clin. Med..

[36] Yasuhara J., Watanabe K., Takagi H., Sumitomo N., Kuno T. (2021). COVID-19 and multisystem inflammatory syndrome in children: A systematic review and meta-analysis. Pediatr. Pulmonol..

[37] Hoste L., Van Paemel R., Haerynck F. (2021). Multisystem inflammatory syndrome in children related to COVID-19: A systematic review. Eur. J. Pediatr..

[38] de Souza T.H., Nadal J.A., Nogueira R.J.N., Pereira R.M., Brandão M.B. (2020). Clinical manifestations of children with COVID-19: A systematic review. Pediatr. Pulmonol..

[39] Gallagher J. (2021). Covid: Which Children are Being Vaccinated and Why?. BBC.

[40] RKI—Empfehlungen der STIKO—Mitteilung der STIKO zur Aktualisierung der COVID-19-Impfempfehlung für Kinder und Jugendliche (16.8.2021). https://www.rki.de/DE/Content/Kommissionen/STIKO/Empfehlungen/PM_2021-08-16.html.

[41] JCVI Statement on COVID-19 Vaccination of Children and Young People Aged 12 to 17 Years: 4 August 2021. https://www.gov.uk/government/publications/jcvi-statement-august-2021-covid-19-vaccination-of-children-and-young-people-aged-12-to-17-years/jcvi-statement-on-covid-19-vaccination-of-children-and-young-people-aged-12-to-17-years-4-august-2021.

[42] JCVI Statement on COVID-19 Vaccination of Children Aged 12 to 15 Years: 3 September 2021. https://www.gov.uk/government/publications/jcvi-statement-september-2021-covid-19-vaccination-of-children-aged-12-to-15-years/jcvi-statement-on-covid-19-vaccination-of-children-aged-12-to-15-years-3-september-2021?fbclid=IwAR1EmXRleP4CkDlOySgOmPXZLnt87E0qZ7AqMZBClrVbqzj1mMOB9jRUDDQ.

[43] Lopez Bernal J., Andrews N., Gower C., Robertson C., Stowe J., Tessier E., Simmons R., Cottrell S., Roberts R., O’Doherty M. (2021). Effectiveness of the Pfizer-BioNTech and Oxford-AstraZeneca vaccines on COVID-19 related symptoms, hospital admissions, and mortality in older adults in England: Test negative case-control study. BMJ.

[44] Cucunawangsih C., Wijaya R.S., Lugito N.P.H., Suriapranata I. (2021). Post-vaccination cases of COVID-19 among healthcare workers at Siloam Teaching Hospital, Indonesia. Int. J. Infect. Dis..

[45] Smith C., Odd D., Harwood R., Ward J., Linney M., Clark M., Hargreaves D., Ladhani S., Draper E., Davis P. (2021). Deaths in Children and Young People in England following SARS-CoV-2 infection during the First Pandemic Year: A National Study using Linked Mandatory Child Death Reporting Data. Research Square.

[46] Saxena S., Skirrow H., Wighton K. (2021). Should the UK vaccinate children and adolescents against covid-19?. BMJ.

[47] Harris R.J., Hall J.A., Zaidi A., Andrews N.J., Dunbar J.K., Dabrera G. (2021). Effect of vaccination on household transmission of SARS-CoV-2 in England. N. Engl. J. Med..

[48] Lu D., Wahlquist C. (2021). Experts say Delta Variant Spread among Australian Children is Concerning in Absence of Covid Vaccine. Guardian.

[49] Children and COVID-19: State-Level Data Report. https://www.aap.org/en/pages/2019-novel-coronavirus-covid-19-infections/children-and-covid-19-state-level-data-report/?fbclid=IwAR0iZOhMtJAVlB25AwO3YsH80D6QvXgSiVpGUTV7sOtEBcq8ZhpxlFlFEQc.

