# Supplementary material for: Attitudes of Parents with Regard to Vaccination of Children against COVID-19 in Poland. A Nationwide Online Survey
Source: Vaccines (Basel). 2021 Oct 17;9(10):1192. doi: 10.3390/vaccines9101192 (PMC8539339; doi:10.3390/vaccines9101192)
Supplement: Supplementary file 1 [file vaccines-09-01192-s001.zip › vaccines-1387867-supplementary.pdf]

Supplementary material - Survey

Attitudes and concerns of parents with regard to vaccinations of children against COVID-19 in Poland.

Dear Sir or Madam,

we are employees of the Department of Family Medicine at the Wroclaw Medical University, Poland, and we would like to analyse parents' attitudes and main concerns towards administering COVID-19 vaccinations in their children.

Accordingly, we invite you to participate in a brief online survey. This survey is fully anonymous and voluntary.

If you have more than one child, please fill out this survey form for each one separately!

Thank you for your willingness to participate.

1. Would you like to take part in the survey?
   Yes/No
2. Do you have children?
   Yes/No
3. How many children do you have?
   a) 1
   b) 2
   c) 3
   d) 4
   e) 5
   f) >5
4. How old is your child?
   a) <6 months
   b) 6 - 23 months
   c) 2-11 years
   d) 12-17 years
5. Does your child have a chronic condition?
   a) Yes
   b) No
6. Sex: Female [ ] Male [ ]
7. Age: ..... years
8. Place of residence:
   a) Rural area
   b) City <50,000 residents
   c) City of 50,000–250,000 residents
   d) City >250,000 residents
9. Education:

   a) Other
   b) Vocational
   c) Secondary education
   d) Higher
10. Are you a healthcare professional?

a) Yes
b) No

11. Have you been vaccinated against COVID-19?
    a) Yes
    b) No, I'm waiting for a vaccination
    c) No, I don't want to get vaccinated
12. If so, did you experience any adverse events after vaccination?
    a) Yes, serious (requiring hospitalisation)
    b) Yes, moderate (e.g. dyspnoea, temperature above 38.5 degrees Celsius, malaise – symptoms lasting more than 72 hours, requiring medical consultation)
    c) Yes, mild (e.g. fever, pain/swelling at the injection site – not requiring medical consultation)
    d) No
13. Have you ever get your children vaccinated according to the immunization schedule?
    a) Yes, only mandatory vaccinations
    b) Yes, both recommended and mandatory vaccinations
    c) No
14. Has your child had adverse postvaccination reactions to any vaccination in the past?
    a) Yes, serious (requiring hospitalisation)
    b) Yes, moderate (e.g. dyspnoea, temperature above 38.5 degrees Celsius, malaise – symptoms lasting more than 72 hours, requiring medical consultation)
    c) Yes, mild (e.g. fever, pain/swelling at the injection site – not requiring medical consultation)
    d) No
15. Have you encountered the COVID-19 vaccination awareness campaign for adults?
    a) Yes
    b) No
16. Have you encountered the COVID-19 vaccination awareness campaign for children and adolescents?
    c) Yes
    d) No
17. On a scale of 1 to 10, how well would you rate your level of knowledge about COVID-19 vaccinations for children and adolescents? (1- I don't know anything, 10 - I have a very high knowledge)
18. What are your sources of information regarding COVID-19 childhood vaccinations? (you can choose more than one option)
    a) The Internet
    b) TV
    c) Medical doctor
    d) Healthcare professionals (other than medical doctors)
    e) Research materials, i.a. scientific journals
    f) Information leaflets
    g) Friends, family (other than healthcare professionals)
    h) Other sources

19. Are you planning to get your child vaccinated against COVID-19?
    a) Yes, as soon as possible
    b) Yes, but in a few months (up to a year)
    c) Yes, but in a year or more
    d) I cannot decide

e) No, but I might consider it in the future
f) No, never

20. On a scale of 1 to 10, how likely are you to get your child vaccinated against COVID-19?
21. Do you think that COVID-19 vaccination should be mandatory for children?
    a) Definitely yes
    b) Rather yes
    c) Neither yes nor no
    d) Rather no
    e) Definitely no
22. Do you think that COVID-19 vaccination should be mandatory for adults?
    a) Definitely yes
    b) Rather yes
    c) Neither yes nor no
    d) Rather no
    e) Definitely no
23. What are your concerns about getting your child vaccinated against COVID-19? (you can choose more than one option)
    a) Adverse events after vaccination
    b) The vaccines have not been sufficiently tested in children
    c) The vaccines are not transported/stored in a proper way
    d) The vaccines might not be effective enough
    e) Others
    f) The vaccines may cause some health problems in the future
    g) I have no concerns
24. On a scale of 1 to 10, how much would you rate COVID-19 as a severe disease for children? (1 - not at all 10 - very severe)
25. On a scale of 1 to 10, how much would you rate that COVID-19 vaccinations are dangerous for children? (1– they are safe, 10 – they are very dangerous)
